# Characterization of Functional Coatings on Cork Stoppers with Laser-Induced Breakdown Spectroscopy Imaging

**DOI:** 10.3390/s23229133

**Published:** 2023-11-12

**Authors:** Miguel F. S. Ferreira, Diana Guimarães, Rafaela Oliveira, Tomás Lopes, Diana Capela, José Marrafa, Pedro Meneses, Armindo Oliveira, Carlos Baptista, Tiago Gomes, Sérgio Moutinho, José Coelho, Raquel Nunes da Silva, Nuno A. Silva, Pedro A. S. Jorge

**Affiliations:** 1 Center for Applied Photonics, INESC TEC, Rua do Campo Alegre 687, 4169-007 Porto, Portugal; miguel.s.ferreira@inesctec.pt (M.F.S.F.); diana.f.guimaraes@inesctec.pt (D.G.); tomas.j.lopes@inesctec.pt (T.L.); diana.f.capela@inesctec.pt (D.C.); nunoazevedosilva@gmail.com (N.A.S.); 2 Departamento de Física e Astronomia, Faculdade de Ciências da Universidade do Porto, Rua do Campo Alegre 687, 4169-007 Porto, Portugal; 3 EGITRON, Rua Central da Vergada, 1280, 4535-166 Mozelos, Portugal; rafaela.oliveira@egitron.pt (R.O.); jose.marrafa@egitron.pt (J.M.); pedro.meneses@egitron.pt (P.M.); armindo.oliveira@egitron.pt (A.O.); 4 Azevedos Indústria, Rua de Santo António 1, Apartado 3, 4536-909 Lourosa, Portugal; cbaptista@azevedosindustria.com (C.B.); tiago@azevedosindustria.com (T.G.); 5 Cork Technological Centre, Rua Amélia Camossa, 4535-368 Santa Maria de Lamas, Portugal; smoutinho@ctcor.com (S.M.); jcoelho@ctcor.com (J.C.); r.sonunesdasilva@gmail.com (R.N.d.S.)

**Keywords:** laser-induced breakdown spectroscopy, cork stoppers, imaging

## Abstract

Evaluating the efficiency of surface treatments is a problem of paramount importance for the cork stopper industry. Generically, these treatments create coatings that aim to enhance the impermeability and lubrification of cork stoppers. Yet, current methods of surface analysis are typically time-consuming, destructive, have poor representativity or rely on indirect approaches. In this work, the use of a laser-induced breakdown spectroscopy (LIBS) imaging solution is explored for evaluating the presence of coating along the cylindrical surface and in depth. To test it, several cork stoppers with different shaped areas of untreated surface were analyzed by LIBS, making a rectangular grid of spots with multiple shots per spot, to try to identify the correspondent shape. Results show that this technique can detect the untreated area along with other features, such as leakage and holes, allowing for a high success rate of identification and for its performance at different depths, paving the way for future industry-grade quality control solutions with more complex surface analysis.

## 1. Introduction

The cork stopper is perhaps the most relevant product resulting from the cork industry, being the most common bottle sealant present in daily life. While cork’s natural properties already provide a good performance in this task [1], manufacturers nowadays may apply surface treatments onto cork stoppers in order to enhance their impermeability and readjust the strength necessary to insert/remove them into/from the bottleneck. Such additional procedures aim to improve the performance of cork stoppers, making it more competitive for the final client. In this context, coating quality is vital to ensure the quality of both the final product and of the consumer experience.

At the production level, cork coating processes are mostly based on batch processing, which can result in an uneven quality distribution. Optimization relies on a posteriori quality assessment using methodologies such as attenuated total reflectance-Fourier transform infrared (ATR-FTIR) spectroscopy, chemical extraction using organic solvents, and measurements of insertion and extraction forces [2,3]. Such methodologies suffer from rather inconvenient drawbacks, those being slow analysis, sample destruction, limited batch representativity and the uncertainty of using indirect methods. Studying and optimizing the treatment process with such limitations is often a complicated task, which can lead to the late detection of a bad batch, requiring the disposal of days’ worth of cork stoppers or a product recall. In worst cases, failing to detect a defective product can lead to losses all the way up the value chain, reaching the consumer. These episodes can result in high economic losses, damaged reputation for all companies involved and even lawsuits [4]. In this context, there is a market need for new methods that can be used for the real-time assessment of coating quality, enabling process control and optimization procedures to be implemented during production.

Laser-induced breakdown spectroscopy (LIBS) is an analytical technique that shows promise in tackling this challenge [5]. It works by analyzing the emission spectra of a cooling plasma that was formed by focusing a pulsed laser into a small area of a sample [6]. This yields almost instant information about the elements comprising the sample that, with more complex analysis, can even be used to classify groups within or between samples across a growing variety of fields [7,8,9]. Besides, the technique itself requires no sample preparation, a significant advantage for this industrial context, making it more suitable for an at-line analysis of a non-planar topology, such as of a cork stopper. Moreover, due to the small spot size of LIBS (down to the µm range), this technique can be used for spectral imaging as it allows for high-resolution pointwise analysis. LIBS imaging has been applied in all sorts of fields such as geology [10,11,12], biology [13,14,15], batteries [16,17], heritage [18] and more [13,19], making it a well-established technique and a strong candidate for the surface analysis of cork stoppers.

In this work, the potential of using a LIBS system as a tool to analyze the quality of functional coatings deposited on cork stoppers is explored for the first time. First, and taking into consideration the specificity of the problem, it is presented a description of the steps of building a custom-based prototype capable of conveniently adapting the LIBS imaging technique to the cylindrical geometry of the sample. Then, to assess the viability and accuracy of this solution, both paraffin- and silicone-based surface treatments, the two most common treatments, were applied to cork stoppers with differently shaped masks, creating shapes of uncoated areas in coated samples. Using the prototype to image elements present at the cork surface, the signal-to-noise ratio (SNR) of specific emission lines (e.g., C and Si) were tracked, which are able to discriminate the coated from the non-coated regions. The presented results showcase the potential of the LIBS imaging technique to assess the coating distribution both on the surface and in depth, paving the way for its further development as a tool for the real-time quality control of functional coatings in the cork industry.

## 2. Materials and Methods

In general terms, LIBS is a technique that uses a focused pulsed laser to ablate a small volume of a sample at its surface and subsequently igniting a plasma. After the end of the laser pulse, the plasma begins to cool down, emitting a continuous background, related to particle collisions and interactions and electron recombinations, and discrete emission lines from ion and atom de-excitations, occurring at specific wavelengths unique to each element. In a typical LIBS system, the emitted light is collected and redirected to a spectrometer to obtain its spectrum. Adding to the system the capability of moving the sample, one can scan the whole surface in pointwise manner (whiskbroom scanning), storing a LIBS spectrum for each point and unlocking the potential of elemental imaging [11,14,17,18].

Motivated by its potential and well-established position in multiple industry-related environments [20], the aim was to explore the viability of using the LIBS imaging technique as a tool to tackle the challenging problem of analyzing the surface treatments applied on cork stoppers. For this purpose, a two-step process was set, starting with the development and deployment of a custom-made LIBS imaging industrial prototype system, while taking costs into account, before establishing a suitable analysis protocol for the cork stopper analysis.

**Figure 1:**
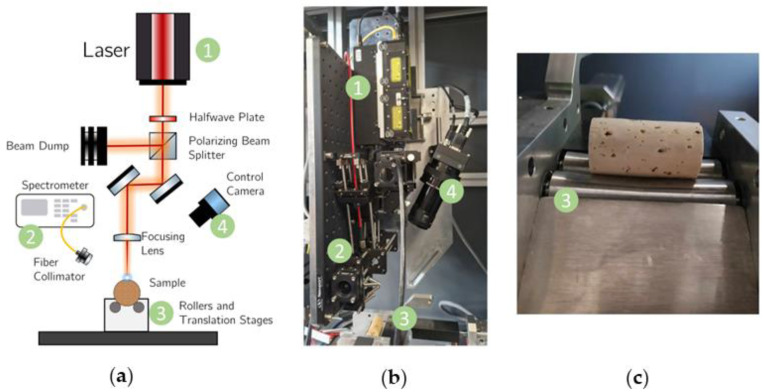
(**a**) Scheme of the LIBS imaging prototype. (**b**) Photo of the prototype and (**c**) highlight of a cork stopper placed on top of the custom-made rollers. 1—Laser source (VIRON laser); 2—Spectrometer (AvaSpec-ULS2048CL-EVO); 3—custom-made rotation system; 4—Line-scan camera (Linea Color CL 4k Color).

Regarding the prototype development stage, a typical LIBS imaging setup was built, as illustrated in Figure 1. In short, a laser source (VIRON laser from LUMIBIRD, Lannion, France, emitting at 1064 nm, 7.3 ns, 50 mJ, and 20 Hz) is utilized in combination with a half-wave plate, polarizing beam-splitter and beam dump, which were used to control the energy of the laser pulse. To ensure versatility and correct alignment, two laser line mirrors steer the laser pulses towards the center of a lens with 150 mm of focal length, leading to an estimated beam waist of 40 µm at the focal spot. To collect the plasma emission in a robust industrial manner, the collection system is comprised of a collimator and optical fiber, connected to a spectrometer from Avantes (AvaSpec-ULS2048CL-EVO, 2048 pixels CMOS detector, Apeldoorn, The Netherlands) in the 220–336 nm spectral range. 

In order to adapt the LIBS imaging to the specific problem of mapping cork samples, a custom-made rotation system using two rollers was developed (Figure 1c). These rollers were designed with a radius that increased from the center to the edges so there is only contact with the samples on their extremities. This avoids as much cross contamination and damage to the coating as possible and enables a repeatable positioning of the sample without significant effort. The rollers rotate the cork stoppers with the help of a motor and stand on top of a horizontal translation stage, which allows for the mapping of the longitudinal direction of a cork stopper and of a vertical translation stage, which allows for the positioning of the cork’s surface close to the focal point of the laser.

Hardware–software integration is achieved using a programmable logic controller (PLC) that controls most of the LIBS imaging process through a program created using TwinCAT 3 (Beckhoff, Verl, Germany), including the rotation/translation stages and the triggers for the laser and the spectrometer. To further synchronize the laser and spectrometer in accurate manners with nanosecond precision, the trigger is split into two signals using a pulse delay generator (PDG) from Amplitude Portugal (Maia, Portugal). A line-scan camera (Linea Color CL 4k Color CMOS Line Scan, Teledyne DALSA, Waterloo, ON, Canada) was also added to the system for visual control of the experiment. Finally, Python-based driver libraries handled the settings for the PDG, laser and spectrometer as well as the data retrieval, treatment and analysis.

**Figure 2:**
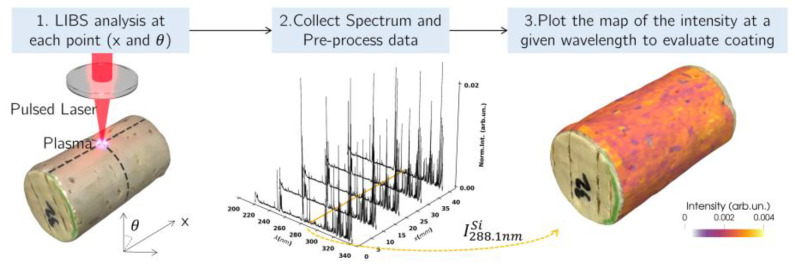
Illustration of the LIBS imaging concept and analysis to perform coating quality evaluations on top of a cork-stopper.

The tested cork stoppers had a length of 45 mm and 24 mm of diameter. As explained in Figure 2, the analysis was performed at the cylindrical face, ignoring 5 mm at each top of the cork stopper to avoid regions contaminated or altered by the manipulation process. Furthermore, to explore the possibility of depth analysis, several consecutive shots were performed at each point, which is known in the literature to provide information related with depth characteristics of samples [16,21,22].

The size of the sampled area and the number of shots per spot were adjusted, taking into consideration the limitations of the maximum number of spectra the spectrometer can save in its RAM. 

For the cork stoppers analyzed, energy was set to around 33 mJ using the half-wave plate and the polarizing beam-splitter with the following qualitative criteria: (i) high intensity spectra; and (ii) saturation prevention. Furthermore, in order to ensure that plasma is not created in air due to the suspension of dust particles from previous shots, the laser repetition rate was set to 10 Hz. Integration time was set to the minimum of the spectrometer (30 µs), and it was opted to have no delay between the start of the integration gate and the laser pulse.

**Figure 3:**
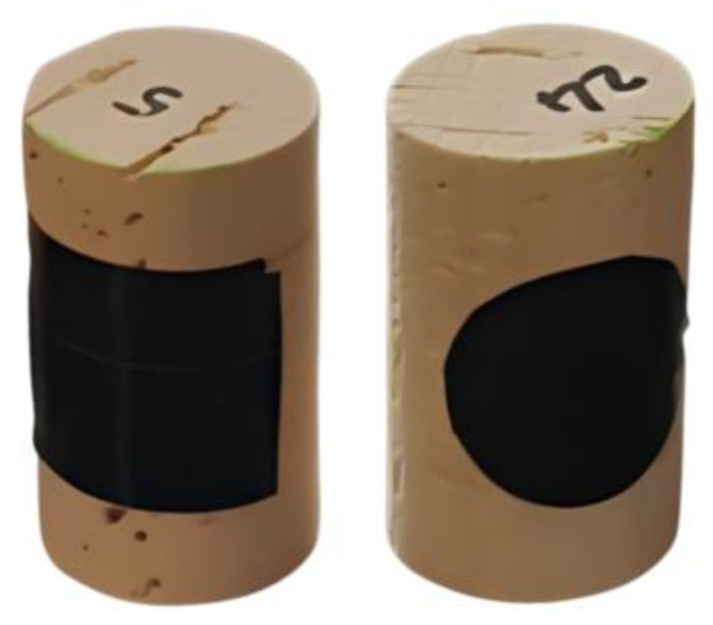
Two examples of masks placed on cork stoppers.

As the major goal of the work was to explore the potential of LIBS imaging for the evaluation of the coating quality of sealant films, simple qualitative tests based on shape-recognition on the surface of cork stoppers were designed. To create such features and facilitate the evaluation of the mapping quality, different geometric-shaped masks were placed on the cork stoppers before applying surface treatments of paraffin and silicone-based materials (Figure 3). These masks were later removed before the LIBS analysis, which were subsequently performed without prior information on the shape of each (i.e., blind tests) to avoid skewing the results unintentionally. This way, the samples tested provided surfaces with known shapes of uncoated areas, allowing for a more efficient evaluation of the LIBS system’s mapping capability.

## 3. Results

**Figure 4:**
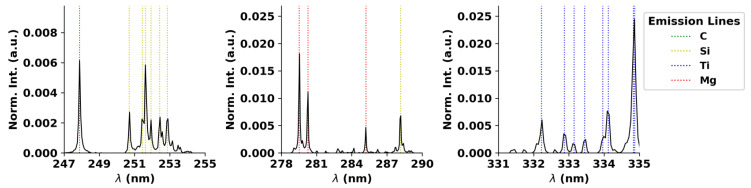
Close ups of an average spectrum of the first shots for a given cork stopper.

All the spectra acquired were subjected to a signal pre-processing. First, using an asymmetric least squares algorithm [23,24], the baselines were extracted from their respective spectra to filter out the continuous background component of the signal. Following the baseline removal, each signal was normalized to the sum of all the intensities in the spectrometer range, which, from literature and empirical observations for this problem, is known to mitigate some of the matrix effects, and it is an alternative to an internal standard [25,26], which could not be used due to the lack of presence of lines of other elements. Figure 4 depicts the main emission lines observed in an average spectrum of a sample.

A quick qualitative analysis shows the presence of emission lines with good SNR for many elements, such as carbon (C), silicon (Si), magnesium (Mg) and titanium (Ti), which validates the quality of the acquired spectrum. Since the final goal is to detect paraffin and silicone-based coatings, the best path to achieve it is to focus on elemental lines, which are exclusive for the coatings and not present in cork itself (*Quercus suber* L.) [27]. From there, images were constructed with the matplotlib python library [28] using the normalized intensity of each selected elemental line integrated with a small radius of 0.2 nm.

### 3.1. Surface Maps

#### 3.1.1. Maps of Silicone Coatings

For silicone-based coatings, one can use Si lines as these are exclusive from the coating itself. Within the range of the spectrometer, many Si-related lines can be found, of which the strongest two, 251.6 and 288.2 nm, were studied. 

**Figure 5:**
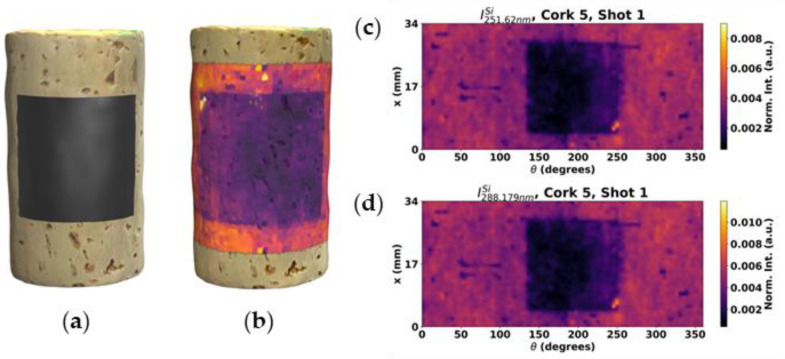
Three dimensional illustrations of the cork stopper 5, with the tape mask before LIBS analysis (**a**) and of the resulting intensity map of the 288.1 nm Si emission line. (**b**) Intensity maps of the Si emission lines at (**c**) 251.6 and (**d**) 288.2 nm.

Figure 5 shows the results obtained for test sample with a squared shaped mask applied before treatment with the functional coating. For this test sample, a 90 × 40 sampling grid of spots evenly distributed around 360° with a step size of 0.85 mm and 4 shots per spots was chosen. In Figure 5c,d, maps obtained for the strongest Si lines can be observed. As expected, both Si line maps yielded very similar results with a small difference in SNR, with both being able to correctly identify the untreated region (corresponding to the shape of the mask). While the mask shape is easily distinguishable, some Si contamination is also visible in the untreated area. Such contamination could have easily occurred due to leakage through surface deformations or gaps due to imperfect mask placement and possible cross surface contamination during storage after the mask removal. 

**Figure 6:**
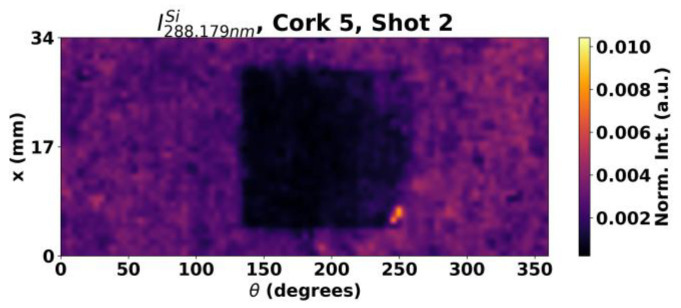
Si map of the second shot in each spatial point on the cork stopper 5.

The latter can be confirmed in Figure 6, where the map obtained with the second shot at each spot can be observed. Because the cross contamination is superficial, it was removed by the first shot, resulting in a map obtained with the second shots showing a cleaner image of the original untreated area.

**Figure 7:**
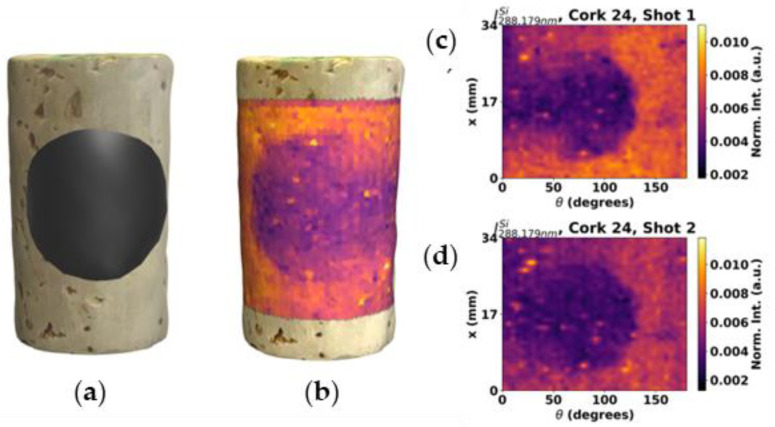
Three dimensional illustrations of the cork stopper 24, with the tape mask before LIBS analysis (**a**) and of the resulting intensity map of the 288.1 nm Si emission line (**b**). Intensity maps of the Si emission lines at 288.2 nm for (**c**) the first and (**d**) second shot.

Using the LIBS technique, 10 out of 12 cork stoppers (Table 1) were successfully mapped using the Si line analysis only. To understand the unsuccessful cases, one is illustrated in Figure 7, for which the analysis was performed using a grid of 45 × 40 spots along 180° with the same step size as the previous case in a region that included the untreated area. As it can be observed in Figure 7c, the circular shape of the masked region can barely be identified at the surface layer. In this and Sample 51’s case, however, a section of the treated area presents a weaker signal than the rest, and it is very similar to the untreated area, suggesting either poor deposition or damage to the silicone coating. Looking at the second shot (Figure 7d), it can be observed that, overall, the signal from the Si line in the treated areas drops in an uneven fashion. This could mean variations in the coating’s thickness. The topic of the thickness of the silicone layer is further explored in Section 3.2.

Overall, and as far as detecting silicone-based surface treatments, LIBS seems to be a suitable technique as it can easily detect the untreated regions, along with some occurring natural defects (such as holes).

#### 3.1.2. Maps of Paraffin Coatings

**Figure 8:**
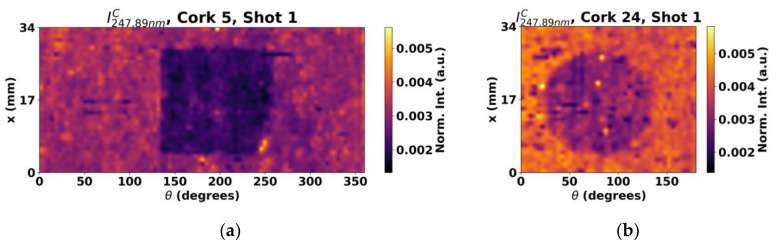
C map of the first shot on the cork stoppers (**a**) 5 and (**b**) 24, using 247.8 nm line.

In the case of paraffin-based coatings, the lack of exclusive elements makes analysis a harder problem to solve. Nevertheless, by exploring the different proportions of the intensity of a given element emission line in cork and in paraffin due to matrix effects, it may be possible to extract information about the treatment quality. Apart from impurities, paraffin is composed of carbon and hydrogen. In the wavelength range of the available spectrometer, there are no H lines, so the focus of the analysis was on the only C line in the range, 247.9 nm. While contrast is lower than that obtained for the silicone case, it is still possible to discern between treated and untreated areas (Figure 8).

Despite the positive result, the lower contrast may anticipate some difficulties in assessing the coating quality when the treatment uniformity and thickness are reduced. 

#### 3.1.3. Exploring Other Elements

**Figure 9:**
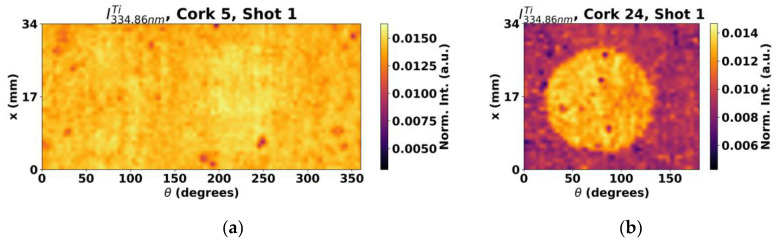
Surface maps obtained by analysis of a Ti^+^ line for (**a**) cork stopper 5 and (**b**) cork stopper 24.

Besides Si and C lines, other lines may contain relevant information about the coating or the cork itself. In this context, some exploratory studies were performed. Such was the case of Ti, which is often present in optional surface color treatments performed on cork stoppers prior to the functional coating application in the form of titanium oxide. Indeed, many Ti^+^ lines were present in the obtained spectra but, for simplicity, the most intense line in the spectral range available, 334.9 nm, was analyzed. The results obtained for cork stopper 5 and 24 can be seen in Figure 9.

In this case, two different outcomes were obtained. In most samples, the shapes from the masks can be clearly identified, as seen in Figure 9b, but now in converse manner, as the intensity signal from the Ti^+^ emission line is now higher at the untreated regions. Such behavior is consistent with the fact that the pigmentation process is applied to the cork surface before the functional coating procedure is carried out. Therefore, the treatment overlay on the surface results in reduced Ti signals in the regions having the functional coating compared to the masked region where the Ti signals are stronger. This way, a very clear picture of the mask features can be obtained analyzing the Ti content. The other case, and sole exception, is presented in Figure 9a. While some of the behavior can be faintly spotted, no shape can be concluded for this case. From analyzing the previous shown maps of Si and C, and by comparing the results from the cork stopper 5 (Figure 5c) and Figure 8a to the ones from cork stopper 24 (Figure 7c and Figure 8b), it is suspected that since their intensity is smaller in cork stopper 5, it contains very thin coatings, having then a weaker effect on the Ti signal.

**Figure 10:**
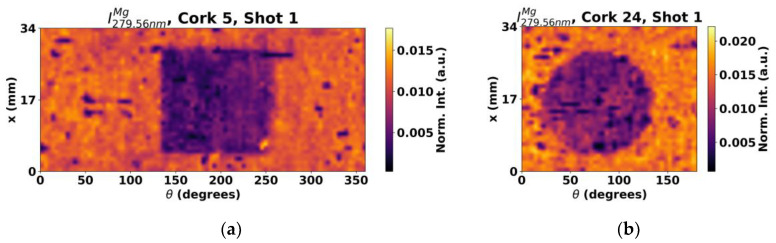
Mg maps of (**a**) cork stopper 5 and (**b**) cork stopper 24.

Magnesium (Mg) is also naturally present in cork as one of the most common elements in mineral form. Figure 10 presents maps for the line 279.6 nm, corresponding to Mg^+^. However, unlike titanium, untreated regions display lower SNR than treated areas and areas affected by cross contamination, suggesting that the surface treatment products also contain magnesium in some form and possibly in a much higher concentration than in the cork stopper.

### 3.2. Depth Analysis

So far, it has been demonstrated qualitatively the ability of a LIBS imaging solution to evaluate coating quality in the presence of extreme coating defects, which were specifically designed for the technology validation. In a more realistic scenario, one would like to use LIBS to assess the overall quality of the surface treatment, evaluating its uniformity. Good coatings in a standard treatment will cover the entire cork stopper in a uniform fashion. Therefore, evaluating the thickness of the coating would be also an interesting perspective for a good industrial solution. In this context, an analysis in depth of the functional coating is desirable, which can be assessed with LIBS technique by using several laser shots at the same spot [16,21,22].

**Figure 11:**
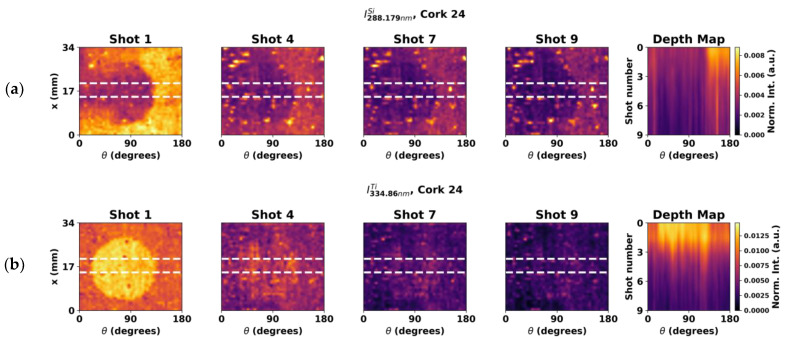
Si and Ti maps of cork stopper 24 using (**a**) 288.2 and (**b**) 334.9 nm lines, respectively, for multiple shots in the same spot. Rightmost column depicting an average intensity map calculated for the highlighted region within the dashed lines on the columns on the left and plotted against the number of shots.

The premise is that for each laser shot a part of the functional coating is ablated and, by applying successive shots, eventually the uncoated cork surface is reached. From the experiments, such behavior was indeed observed, as can be seen in Figure 11. Here, the normalized intensity of the Si lines on the treated area decreases with successive shots, eventually converging to the similar values obtained in the untreated area after several shots, indicating that all the coating was eventually ablated. A more pronounced yet similar surface effect can be observed in the Ti intensity maps shown in Figure 11b.

Overall, both these results clearly indicate the possibility of using LIBS imaging to evaluate the uniformity of the coating not only at the surface but also in relation to thickness, paving the way for future research in this topic.

## 4. Discussion

The results enclosed in this manuscript show promising results for LIBS as a suitable technique for evaluating the quality of surface treatments in cork stoppers.

On one hand, regarding different coating types, it was first observed that Si lines are easily detected, thus presenting a reliable way to map silicone-based coatings. Paraffin on the other hand might require better system specification (e.g., more sensitive spectrometer), parameter optimization (e.g., higher pulse energy) or even a more complex data analysis to discern. Indeed, although the results suggest that it is possible to map paraffin through the use of a C line, they also suggest that sensitivity might be worse due to the presence of carbon in both the cork stoppers and paraffin.

On the other hand, in addition to these specific elements, the potential of evaluating the surface treatment through indirect methodologies was also observed. In specific, it was found that the intensity of the Ti emission lines is higher in untreated areas. Although it cannot be said that such emission line establishes a reliable way to evaluate the quality of coatings, it is still worth additional exploration in future studies due its apparent immunity to cross contamination. In a similar perspective, Mg analysis might also be an interesting possibility if a surface treatment product contains this element, as it can be used as a mean to track down the product as a whole.

Timewise, LIBS fares well against current methods. Depending on the technique and coating, they can range from 2 to 24 h per sample. The LIBS prototype took around 2 h to complete the largest map grid (cork stopper number 5), which is an extremely overdetailed analysis of the sample. For a full industry implementation, the number of analyzed spots is expected to be reduced to fewer than 100, decreasing dramatically the time for each sample.

Lastly, by digging through the coatings using multiple shots per spot, it is visible that the normalized intensity of Si starts to decay with the number of shots, eventually becoming similar to that of a region with no surface treatment. This finding suggest that it is possible to analyze the thickness of the coating by studying the intensity of the emission lines of chemical elements present in its composition at increasing depths.

## 5. Conclusions

In this manuscript, the capabilities of LIBS to provide insight into the quality of functional coatings in cork stoppers were tested for the first time. A prototype customized to properly manage the cylindrical geometry and the handling constraints of the coated stoppers was implemented. A methodology to assess the coating distribution along the surface and in depth was developed based on the mapping of distinct elements, exclusive to either the coating or the cork, or present in both, but having distinct proportions.

From the depth analysis, results show viability to identify heterogeneities on the surface and evaluate the thickness of the coating, which urges future research. The fine-tuning of the instrumentation specs, operational parameters and processing methodologies will further improve the performance, setting LIBS as the basis for a unique quality and process control tool of the coating processes, potentially improving the quality and reliability of the final product, benefiting both the industry and the customer experience.

## 6. Patents

One Portuguese patent resulted from the work reported in this manuscript (PT116928A).

## Figures and Tables

**Table 1:** List of samples, respective masks and success rate of LIBS.

Sample	Shape	Identified (Y/N)
1	Square	Y
5	Square	Y
16	Triangle	Y
17	Triangle	Y
22	Circle	Y
24	Circle	N
25	Circle	Y
37	Parallelogram	Y
46	Non-regular heptagon	Y
51	Cross	N
52	Cross	Y
53	Cross	Y

## Data Availability

Data analyzed in this manuscript may be shared upon reasonable request.

## References

[1] Silva S.P., Sabino M.A., Fernandes E.M., Correlo V.M., Boesel L.F., Reis R.L. (2005). Cork: Properties, capabilities and applications. Int. Mater. Rev..

[2] Ortega-Fernández C., González-Adrados J.R., García-Vallejo M.C., Calvo-Haro R., Cáceres-Esteban M.J. (2006). Characterization of surface treatments of cork stoppers by FTIR-ATR. J. Agric. Food Chem..

[3] Gonzalez-Adrados J.R., Garcia-Vallejo M.C., Caceres-Esteban M.J., Garcia De Ceca J.L., Gonzalez-Hernandez F., Calvo-Haro R. (2012). Control by ATR-FTIR of surface treatment of cork stoppers and its effect on their mechanical performance. Wood Sci. Technol..

[4] Todorov K. Cayuse’s Insurance Company Sues Lafitte for Allegedly Selling Defective Corks. https://www.winebusiness.com/news/article/225210.

[5] Veloso H.A. (2020). Optimization of Laser-Induced Breakdown Spectroscopy (LIBS) for Application in the Cork Industry. Master’s Thesis.

[6] Cremers D.A., Radziemski L.J. (2013). Handbook of Laser-Induced Breakdown Spectroscopy.

[7] Capela D., Ferreira M.F.S., Lima A., Dias F., Lopes T., Guimarães D., Jorge P.A.S., Silva N.A. (2023). Robust and interpretable mineral identification using laser-induced breakdown spectroscopy mapping. Spectrochim. Acta Part B At. Spectrosc..

[8] Feng J., Wan E., Han B., Chen Z., Liu X., Liu Y. (2023). Research on identification of ink marks based on machine learning and laser-induced breakdown spectroscopy. J. Laser Appl..

[9] Chen Z., Zhai R., Cai Y., Ye Y., Sun Z., Liu Y. (2023). Online source tracing of waste paper by smoke based on laser-induced breakdown spectroscopy. J. Laser Appl..

[10] Trichard F., Moncayo S., Devismes D., Pelascini F., Maurelli J., Feugier A., Sasseville C., Surma F., Motto-Ros V. (2017). Evaluation of a compact VUV spectrometer for elemental imaging by laser-induced breakdown spectroscopy: Application to mine core characterization. J. Anal. At. Spectrom..

[11] Fabre C., Devismes D., Moncayo S., Pelascini F., Trichard F., Lecomte A., Bousquet B., Cauzid J., Motto-Ros V. (2018). Elemental imaging by laser-induced breakdown spectroscopy for the geological characterization of minerals. J. Anal. At. Spectrom..

[12] Ribeiro R., Capela D., Ferreira M., Martins R., Jorge P., Guimarães D., Lima A. (2021). X-ray fluorescence and laser-induced breakdown spectroscopy analysis of Li-rich minerals in veins from argemela tin mine, central Portugal. Minerals.

[13] Trichard F., Sorbier L., Moncayo S., Blouët Y., Lienemann C.P., Motto-Ros V. (2017). Quantitative elemental imaging of heterogeneous catalysts using laser-induced breakdown spectroscopy. Spectrochim. Acta Part B At. Spectrosc..

[14] Moncayo S., Trichard F., Busser B., Sabatier-Vincent M., Pelascini F., Pinel N., Templier I., Charles J., Sancey L., Motto-Ros V. (2017). Multi-elemental imaging of paraffin-embedded human samples by laser-induced breakdown spectroscopy. Spectrochim. Acta Part B At. Spectrosc..

[15] Gimenez Y., Busser B., Trichard F., Kulesza A., Laurent J.M., Zaun V., Lux F., Benoit J.M., Panczer G., Dugourd P. (2016). 3D Imaging of Nanoparticle Distribution in Biological Tissue by Laser-Induced Breakdown Spectroscopy. Sci. Rep..

[16] Kappeler M., Basler C., Brandenburg A., Carl D., Wöllenstein J. (2022). Homogeneity Measurements of Li-Ion Battery Cathodes Using Laser-Induced Breakdown Spectroscopy. Sensors.

[17] Baptista M.C., Gomes B.M., Capela D., Ferreira M.F.S., Guimarães D., Silva N.A., Jorge P.A.S., Silva J.J., Braga M.H. (2023). Conditioning Solid-State Anode-Less Cells for the Next Generation of Batteries. Batteries.

[18] Richiero S., Sandoval C., Oberlin C., Schmitt A., Lefevre J.-C., Bensalah-Ledoux A., Prigent D., Coquidé C., Valois A., Giletti F. (2022). Archaeological Mortar Characterization Using Laser-Induced Breakdown Spectroscopy (LIBS) Imaging Microscopy. Appl. Spectrosc..

[19] Alombert-Goget G., Trichard F., Li H., Pezzani C., Silvestre M., Barthalay N., Motto-Ros V., Lebbou K. (2017). Titanium distribution profiles obtained by luminescence and LIBS measurements on Ti: Al_2_O_3_ grown by Czochralski and Kyropoulos techniques. Opt. Mater..

[20] Jolivet L., Leprince M., Moncayo S., Sorbier L., Lienemann C.P., Motto-Ros V. (2019). Review of the recent advances and applications of LIBS-based imaging. Spectrochim. Acta Part B At. Spectrosc..

[21] Mowery M.D., Sing R., Kirsch J., Razaghi A., Béchard S., Reed R.A. (2002). Rapid at-line analysis of coating thickness and uniformity on tablets using laser induced breakdown spectroscopy. J. Pharm. Biomed. Anal..

[22] Wan E., Tian D., Sun Z., Liu Y. (2023). The online in situ detection of plastic and its combustion smoke via laser-induced breakdown spectroscopy. Spectrosc. Lett..

[23] Eilers P.H.C. (2003). A perfect smoother. Anal. Chem..

[24] Eilers P.H.C. (2004). Parametric Time Warping. Anal. Chem..

[25] Guezenoc J., Gallet-Budynek A., Bousquet B. (2019). Critical review and advices on spectral-based normalization methods for LIBS quantitative analysis. Spectrochim. Acta Part B At. Spectrosc..

[26] Zorov N.B., Gorbatenko A.A., Labutin T.A., Popov A.M. (2010). A review of normalization techniques in analytical atomic spectrometry with laser sampling: From single to multivariate correction. Spectrochim. Acta Part B At. Spectrosc..

[27] Pereira H. (1988). Chemical composition and variability of cork from *Quercus suber* L.. Wood Sci. Technol..

[28] Matplotlib: Visualization with Python. https://matplotlib.org/.

